# Insights into the phylogenetic and metabolic diversity of *Planctomycetota* in anaerobic digesters and the isolation of novel *Thermoguttaceae* species

**DOI:** 10.1093/femsec/fiaf025

**Published:** 2025-03-17

**Authors:** Dominika Klimek, Malte Herold, Inês Rosado Vitorino, Zuzana Dedova, Sebastien Lemaigre, Jimmy Roussel, Xavier Goux, Olga Maria Lage, Magdalena Calusinska

**Affiliations:** Environmental and Industrial Biotechnology, Luxembourg Institute of Science and Technology (LIST), L-4970 Hautcharage, Luxembourg; Faculty of Science, Technology and Medicine (FSTM), University of Luxembourg, L-4364 Esch-sur-Alzette, Luxembourg; Environmental and Industrial Biotechnology, Luxembourg Institute of Science and Technology (LIST), L-4970 Hautcharage, Luxembourg; Department of Biology, Faculty of Sciences, University of Porto, 4169-007 Porto, Portugal; Environmental and Industrial Biotechnology, Luxembourg Institute of Science and Technology (LIST), L-4970 Hautcharage, Luxembourg; Environmental and Industrial Biotechnology, Luxembourg Institute of Science and Technology (LIST), L-4970 Hautcharage, Luxembourg; Environmental and Industrial Biotechnology, Luxembourg Institute of Science and Technology (LIST), L-4970 Hautcharage, Luxembourg; Environmental and Industrial Biotechnology, Luxembourg Institute of Science and Technology (LIST), L-4970 Hautcharage, Luxembourg; Department of Biology, Faculty of Sciences, University of Porto, 4169-007 Porto, Portugal; Interdisciplinary Centre of Marine and Environmental Research (CIIMAR/CIMAR), 4450-208 Matosinhos, Portugal; Environmental and Industrial Biotechnology, Luxembourg Institute of Science and Technology (LIST), L-4970 Hautcharage, Luxembourg

**Keywords:** *Planctomycetota*, *Planctomycetes*, anaerobic digestion, encoded metabolic potential, CAZymes, bacterial utilization of sulphated glycans, mucins, algal polysaccharides, exoglycosidases

## Abstract

Studying bacteria in anaerobic digestion (AD) is crucial for optimizing microbial processes. While abundant taxa are often studied, less abundant groups may harbour novel metabolic potential. This study fills the gap by focusing on the *Planctomycetota* phylum, known to encode diverse carbohydrate-active enzymes (CAZymes). Despite their common presence in diverse aerobic and anaerobic environments, their role in AD is relatively unexplored. We utilized both culture-dependent and culture-independent techniques to investigate the phylogenetic and metabolic diversity of *Planctomycetota* within AD reactors. Our findings revealed that among the diverse planctomycetotal operational taxonomic units present, only a few are prevalent and abundant community members. *Planctomycetota* share functional traits with e.g. *Verrucomicrobiota* exhibiting distinct CAZyme gene repertoires that indicates specialization in degrading algal polysaccharides and glycoproteins. To explore the planctomycetotal metabolic capabilities, we monitored their presence in algal-fed digesters. Additionally, we isolated a strain from mucin-based medium, revealing its genetic potential for a mixotrophic lifestyle. Based on the genomic analysis, we propose to introduce the *Candidatus* Luxemburgiella decessa gen. nov. sp. nov., belonging to the *Thermoguttaceae* family within the *Pirellulales* order of the *Planctomycetia* class. This study enhances our understanding of *Planctomycetota* in AD by highlighting their phylogenetic diversity and metabolic capabilities.

## Teaser

Strain of a novel genus within the *Thermoguttaceae* isolated from the rare biosphere *Planctomycetota* of an anaerobic digester.

## Introduction

The anaerobic digestion (AD) process is commonly used to produce biogas, of which methane is the energy source (Kougias and Angelidaki 2018). Methane can be upgraded to natural gas quality or biogas can be burned to generate heating and electricity (Angelidaki et al. 2018). Biogas is produced via the microbial decomposition of organic matter, such as agricultural and food waste, wastewater-activated sludge, or even seaweed, and its yield is directly influenced by microbial activities in the digesters (Hughes et al. 2012, Lim et al. 2020, Calbry-Muzyka et al. 2022). Understanding microbial metabolic capacities and limitations can optimize the conditions to maximize the biogas yield (Blair et al. 2021). Additionally, maintaining a balanced microbial community is crucial for ensuring a stable process and preventing failures and inefficiencies (Goux et al. 2015, Blair et al. 2021, Lemaigre et al. 2023). Given this, research in this area is not only important but rather essential for advancing and optimizing the development of the green energy sector (Carballa et al. 2015).

Advances in metagenomics have enabled the reconstruction of microbial genomes directly from environmental samples, providing new insights into the genetic potential of the previously unknown taxa (Stewart et al. 2019). Metabolic predictions based on genomic data allow us to infer the biological processes in which bacteria might be engaged, suggesting their roles in the environment (Anantharaman et al. 2016). Metabolic modelling can also partially enhance isolation efforts through reverse-genomic culturing approaches (Cross et al. 2019). Microbial communities in different environments encompass an immense diversity with a significant fraction of so-called rare biosphere (Pedrós-Alió 2012). Some rare taxa are transient bacteria or on the brink of extinction, while others may remain dormant awaiting favourable conditions (Shade et al. 2014). Nevertheless, it has been shown that in many habitats, rare or low-abundant bacteria are critical keystone taxa that ensure the proper functioning of microbial community, despite their disproportionate abundance (Pascoal et al. 2020). The microbial activity of the rare biosphere is linked to its buffering capacity with the potential to mitigate the effects of disturbances, often possessing unique metabolic capacities (Lynch and Neufeld 2015, Zhao et al. 2024). The activity of rare taxa in diverse environments can be linked to pollutant degradation and higher lignocellulose degradation rates, as well as sulphate reduction and denitrification, to name but a few (Jousset et al. 2017, Wang et al. 2017, Puentes-Téllez and Salles 2020). Likewise, the scarce bacteria may exert a significant impact on the overall AD process and even become dominant, depending on the bioreactor operation conditions (Dueholm et al. 2023). For instance, the low-abundant syntrophic acetate or propionate oxidizing bacteria play a critical role in the conversion of acetate and propionate, living in symbiosis with methanogens for hydrogen and carbon dioxide removal (Westerholm et al. 2022, Singh et al. 2023).

Among the diverse phyla present in AD systems operating across the globe (Centurion et al. 2024), *Planctomycetota*, previously known as *Planctomycetes* (Oren and Garrity 2021), seems to be an overlooked group of microorganisms. This phylum encompasses unique bacteria known for their distinctive cell biology, including an enlarged periplasm, unusual structures, and FtsZ-independent divisome mode (Lage et al. 2013, Boedeker et al. 2017, Rivas-Marin et al. 2020). While diverse members of *Planctomycetota* are present in various habitats including soil, freshwater, and marine ecosystems (Wiegand et al. 2020), their abundance is relatively low but persistent in the AD system (Campanaro et al. 2020). According to the collective microbial diversity in ADs, *Planctomycetota* were categorized as sporadically occurring bacteria (Nelson et al. 2011), although certain representatives have sometimes been reported to be highly enriched in the different AD settings (Zheng et al. 2015, Hailu et al. 2021). Still, they are typically discussed in the context of nitrogen removal since the anaerobic ammonium oxidation (anammox) bacteria are specialized in utilizing ammonium and nitrite under anaerobic conditions (Yangin-Gomec et al. 2017, Bellucci et al. 2022). However, anammox bacteria, which mainly belong to the *Candidatus* Brocadiia class (previously *Ca*. Brocadia), are physiologically distinct from other known *Planctomycetota* such as *Planctomycetia* and *Phycisphaerae* (Kartal et al. 2012). Based on metaproteomic and metagenomic analyses, *Planctomycetota* were identified as bacteria with high hydrolytic potential due to the presence of multiple carbohydrate-active enzymes (CAZymes) (Vanwonterghem et al. 2016, Villalobos Solis et al. 2022, Klimek et al. 2024). This observation places *Planctomycetota* as overlooked but important biomass degraders in the AD environment. The *Sedimentisphaerales* order of the *Phycisphaerae* class was identified as a widespread lineage in anaerobic habitats (Spring et al. 2018) while the high diversity of *Planctomycetia* was found in wastewater ADs (Chouari et al. 2003). Axenically isolated strains of *Planctomycetota* are primarily aerobic and derive from marine and freshwater environments (Wiegand et al. 2020). To date, only one publicly known *Planctomycetota* strain has been isolated from anaerobic digesters, *Thermopirellula anaerolimosa* (Liu et al. 2012), highlighting the need for focused isolation studies from anoxic and underexplored environments.

In this study, we explore the potential role of *Planctomycetota*, which we consider a member of the rare biosphere within the AD environment. We elucidate their phylogenetic and metabolic diversity through targeted and shotgun metagenomic approaches. We next describe their genomic characteristics and potential metabolic capabilities, providing new insights into their ecological roles in AD systems. Guided by these results, we elaborate a set of potential growth promoters to specifically isolate *Planctomycetota* from the ADs operated with different feedstocks. Based on the phylogenetic reconstruction, we propose that the isolated strain constitutes a novel species and genus within the *Thermoguttaceae* family of the *Pirellulales* order.

## Materials and methods

### Sample processing, 16S rRNA sequencing, and analysis

For the 16S rRNA gene amplicon analysis, we relied on the set of samples from the previous study of coauthors (Calusinska et al. 2018). Briefly, the sludge samples were collected over the year from different AD reactors located in ten full-scale AD units (U3–U10; Supplementary File 1, Table S1) in Belgium and Luxembourg. The ‘U’ category denotes the sampling location, however, not all reactors studied included *Planctomycetota* in the bacterial community. The AD reactors were fed with different types of feedstocks and were classified into the following categories: (1) farm AD (agriculture residues and biowaste; U3–U5), (2) OFMSW (ADs supplied with the organic fraction of the municipal solid waste; U7), and (3) WWTP ADs (sewage sludge from wastewater treatment plant; U8–U10). DNA was extracted with the QIAGEN PowerSoil Kit and 16S rRNA sequences were amplified (amplicon size about 484 bp) and sequenced on the Illumina Miseq platform using MiSeq Reagent Kit V3-600 cycles. In this study, we only reanalysed the generated data. The raw sequencing output reads were merged, demultiplexed and trimmed (minimum length of 400 bp) using usearch v11.0.667. Operational taxonomic units (OTUs) were clustered at 3% difference and their sequences were taxonomically annotated using mothur v1.48.0 (Schloss et al. 2009) against the nonredundant SILVA SSU database v138 (Quast et al. 2013). At this step, only reads assigned to *Planctomycetota* (100) were retained (Supplementary File 1, Table S1). The OTU sequences were additionally blasted against a custom database containing the 16S rRNA gene sequences of all the type strains of *Planctomycetota* to date. For this purpose, we downloaded 148 16S rRNA gene sequences from the ‘type material’ NCBI database (accessed June 2024), which is a collection of the reference isolated and described strains. The downstream analyses were performed in R v4.4.0. The phylogenetic tree of planctomycetotal OTUs was built in *Geneious* v2019.0.3, by aligning the OTU sequences with the MUSCLE aligner for constructing trees using the Neighbour-Joining algorithm with default parameters. To evaluate the performance of the primer set used, we ran *in silico* polymerase chain reaction (PCR) using TestPrime 1.0 available on the https://www.arb-silva.de/search/testprime/ website (Supplementary File 1, Table S2).

### Sample processing, novel *Planctomycetota* MAGs, and functional genomic analyses

In order to describe the metabolic potential of *Planctomycetota*, we collected AD-sourced planctomycetotal genomes from our previous analysis (Klimek et al. 2024) and further extended this dataset with genomes assembled in this study, isolated from one enrichment culture and sludge inocula. For the latter, two samples were collected for metagenome reconstruction from two reactors located in Luxembourg: the WWTP in Bettembourg and a private operational unit fed with agriculture residues and biowaste. DNA was extracted using the QIAGEN PowerSoil Kit and was sent to the University of Luxembourg for sequencing. The sequencing was performed using NextSeq2000 Illumina platform with P3 flowcell 300 cycles. The raw sequence output was processed with the MuDoGeR pipeline v1.0.1 (Rocha et al. 2024) utilizing module-1 for preprocessing including adapter trimming with trim-galore v0.6.7 and metagenomic assembly with MEGAHIT v1.2.9 (Li et al. 2016) and metaSPAdes v.3.15.5 (Nurk et al. 2017). The resulting final assembly from MuDoGeR (joined file of the long contigs generated with both assemblers) was used as the input for sample-wise metagenomic binning with SemiBin v1.5.1 (Pan et al. 2022). To generate input mappings, reads were mapped to the final assembly with Bowtie2 v2.2.5 (Langmead and Salzberg 2012). Binning was performed with the single_easy_bin workflow and ‘self’ training type, resulting in 312 medium quality metagenome-assembled genomes (MAGs with >50% completeness, <10% contamination). Of all the assembled MAGs, 14 were assigned to *Planctomycetota* with half representing new genome-species based on genome comparisons using FastANI v1.31. This set, along with the planctomycetotal genomes collected from public and our own repositories, resulted in the creation of a database containing 38 nonredundant medium- to high-quality genomes of the AD *Planctomycetota* (Supplementary File 2, Table S3). The genomes were dereplicated using dRep v3.3.0 with default parameters to obtain the representative genomes (Olm et al. 2017). Subsequently, genomes were gene-called using prodigal and annotated with Prokka v1.14.6 to obtain clusters of orthologous genes entries (Hyatt et al. 2010, Seemann 2014). Kyoto Encyclopaedia KEGG were attained with KO terms using the online KEGG blast koala tool, following which, KO entries were mapped into putative pathways and functional traits using the KEGG mapper and microtrait tools (Kanehisa et al. 2016, 2023, Karaoz and Brodie 2022). The genomes were additionally annotated for CAZymes using the dbCAN3 online server (Drula et al. 2022). To predict the potential peptidases and sulphatases, a blast search was run against the MEROPS and SulfAtlas databases (Rawlings et al. 2014, Stam et al. 2023). To classify the catalytic subunits of predicted hydrogenases, we used the online HydDB tool and database (Søndergaard et al. 2016). To identify the genes coding for iron cycling we used FeGenie (Garber et al. 2020).

### Genome-guided enrichment and isolation of *Planctomycetota*

To evaluate the specific role of *Planctomycetota* in AD systems, we compared their metabolic potential encoded in the *Planctomycetota* database to that of other AD microbes characterized in (Campanaro et al. 2020), which represents the most comprehensive database available at the time of analysis. The downloaded genomes were further annotated with KO and CAZyme entries for subsequent comparative genome analysis, which in continuation guided our isolation attempts. A linear discriminant Effect Size analysis implemented in mothur v1.48.0 was used to verify the differentially encoded genes in *Planctomycetota* genomes. Based on the predicted genomic features, a set of culture media was designed to target and enhance the growth of *Planctomycetota* specifically. The exact composition of media and enrichment conditions is available in Supplementary File 3, Table S7. Briefly, enrichments were performed in 120 ml serum bottles with working volume of 20–50 ml. The initial pH was set to between 5 and 7 and the temperature between 20°C and 37°C. Different basal media and a set of various carbon and nitrogen sources were used. The inoculum consisted of anaerobic sludge taken from the WWTP (Bettembourg, Luxembourg) or the private AD (agriculture biowaste) operational units at the different timepoints. Before inoculation, samples were mixed well and treated either with antibiotics and/or FtsZ-stabilizers (Supplementary File 3, Table S7). The enrichments were subsequently seeded with 0.1%–10% (v/v) of pretreated sludge. The incubation time was between 2 and 8 weeks. At the end of the given incubation time, DNA was isolated using the QIAGEN PowerSoil Kit for 16S rRNA gene data sequencing on the preselected samples. Libraries were sequenced on either the Illumina or Oxford Nanopore systems. Illumina sequencing was prepared according to the aforementioned procedure (‘Sample processing, 16S rRNA sequencing and analysis’ paragraph). For Oxford Nanopore sequencing, culture lysate was prepared and sequenced on the MinION flow cell R9.1 (SQK-16S024 library prep) until a minimum of 50k reads were sequenced. Upon obtaining the 16S rRNA data analysis results, selected samples were further purified using diverse approaches, including for instance dilution-to-extinction or iChip-inspired methods (detailed in Supplementary File 3, Tables S7–S10) (Vitorino et al. 2021).

### Genome sequencing and analysis of isolated strains of *Planctomycetota*

SKZ1R and SKZ5 enrichment samples were selected for genome sequencing. The DNA of the SKZ1R culture was isolated using an in-house chloroform–isopropanol DNA extraction method and precipitated overnight in ethanol containing 0.02 M sodium acetate. DNA sequencing was performed on Illumina and Nanopore (SQK-LSK109 library prep) systems as described above. A hybrid genome was assembled using Flye v2.8.1 assembly on Nanopore reads (Kolmogorov et al. 2019) and polished with Illumina reads using nanopolish v0.13.2. The genome quality check was measured by both checkM v1.2.0 and checkM2 v (Chklovski et al. 2023, Parks et al. 2015). For a comparison, the genome was functionally annotated with Prokka (Seemann 2014), blastKOALA (Kanehisa et al. 2016), and Rapid Annotations Using Subsystems Technology (RAST) server (Aziz et al. 2008). Protein sequences were additionally annotated for Pfam domains using the HMMER (Eddy 2011) against the Pfam HMM database. The DNA of enrichment of SKZ5 was isolated using PowerSoil QIAGEN and was only sequenced on the Illumina platform. To retrieve *Planctomycetota* genomes from the SKZ5 enrichment, pipeline mags-nextflow (https://nf-co.re/mag/2.5.1) was utilized with default parameters (Ewels et al. 2020). For SKZ5 enrichment, two assemblies were prepared: MEGAHIT and SPAdes (Li et al. 2016, Prjibelski et al. 2020) and binning results from METABAT2 were further dereplicated using dRep (Kang et al. 2019, Olm et al. 2017). The nonredundant bins were assigned taxonomically by GTDB-tk against GTDB database (Chaumeil et al. 2020, Parks et al. 2018) and genome annotation was conducted as for the SKZ1R strain. To infer the novelty of reconstructed genomes, average aminoacid identity (ANI, OrthoANIu) and average aminoacid identity (AAI) values were calculated using ANI.jar and enveomics.aai scripts, respectively (Rodriguez-R and Konstantinidis 2016, Yoon et al. 2017). 16S rRNA phylogeny was inferred for the family-level type strain representative sequences within *Pirellulales*, retrieved from the NCBI GenBank. The genome of *Planctopirus limnophila* DSM 3776 was used as an outgroup for tree reconstruction (NR_074670.1). To expand the phylogenetic analysis, a set of MAGs assigned to *Thermoguttaceae* in GTDB was downloaded from the NCBI GenBank using the genome_updater.sh script from https://github.com/pirovc/genome_updater and was dereplicated with dRep at 99% ANI (Olm et al. 2017). After quality checking, a phylogeny of 129 nonredundant genomes was built on the translated coding sequences using phylophlan with—diversity high parameter and the tree was further annotated and visualized by iTOL (Asnicar et al. 2020, Letunic and Bork 2021). Another phylogenetic marker, the beta subunit of the bacterial RNA polymerase coding gene (rpoB), was extracted from the studied genomes and compared to the rpoB gene of *Thermogutta terrifontis* R1, the only sequenced representative of the family *Thermoguttaceae*.

### Algal biomass degradation in a BMP test

The biochemical methane potential (BMP) test was performed in triplicates using the Automatic Methane Potential Test System (AMPTS II, Bioprocess Control). Three types of biomasses were used: cyanobacterial powder of *Aphanizomenon* flos-aquae, macroalgal powder of *Macrocystis pyrifera* (giant kelp), and sugar beet pulp (SBP) powder as a control biomass. 50 ml of sludge originating from the WWTP AD reactor was used as the inoculum, and it was then fed on cellulose for 10 days to acclimatize its microbiome for laboratory conditions. The experiment lasted 40 days, with a constant temperature of 37°C, and daily mixing and methane gas measurements. For microbial community analysis, the representative samples of each replicate condition were selected for DNA extraction and collected on the first and 40th day of the experiment. The sequencing was performed using Nanopore sequencing. The library was prepared using an SQK-16S024 kit and sequenced on a MinION flow cell R9.1. The sequence data were processed using the epi2me-labs/wf-16 s nextflow workflow (https://github.com/epi2me-labs/wf-16 s).

## Results

### Phylogenetic diversity of *Planctomycetota* in ADs based on 16S rRNA gene sequencing and SILVA taxonomy

**Figure 1.**
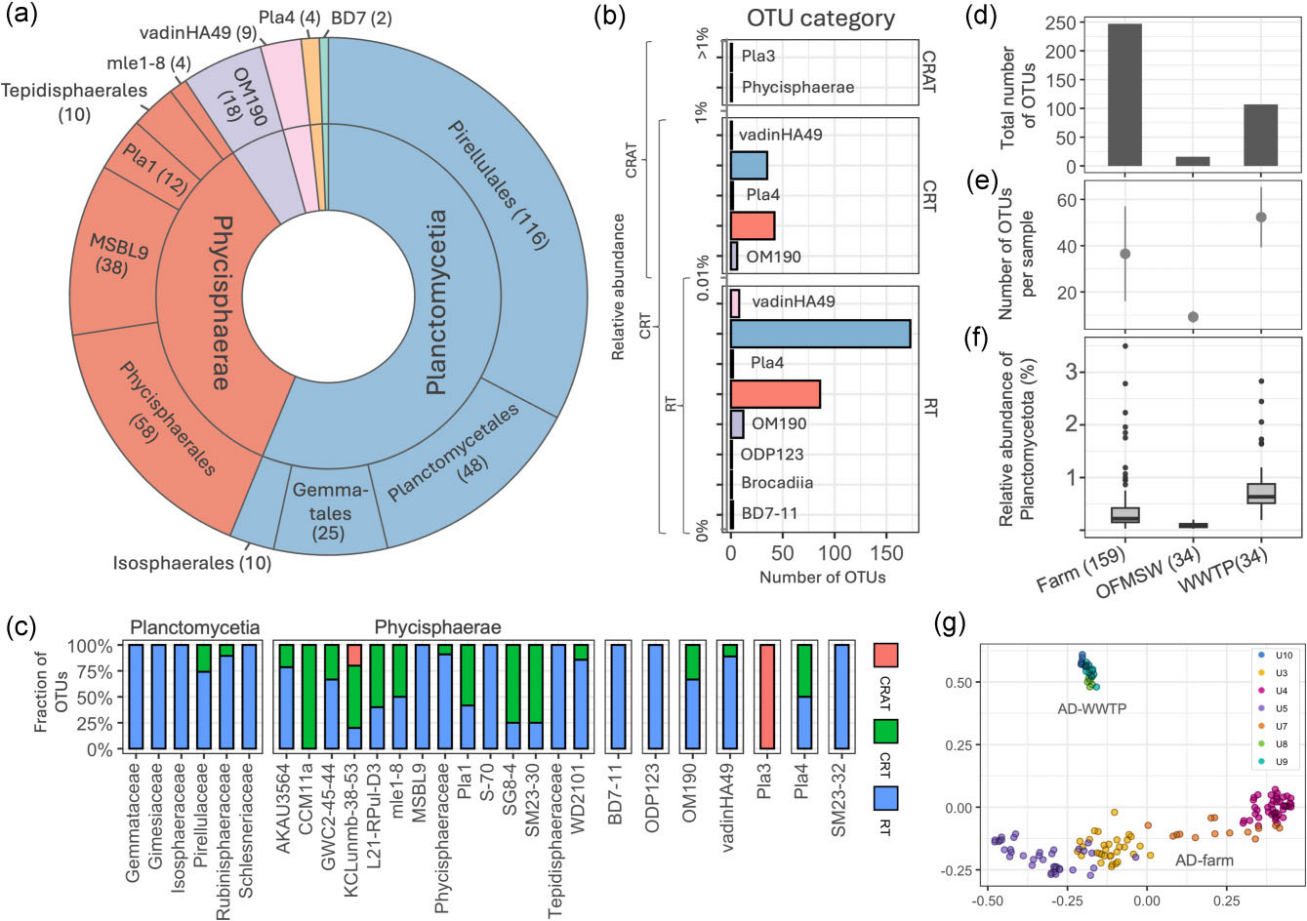
Planctomycetotal OTU diversity based on the 16S rRNA gene amplicon sequencing and SILVA taxonomy. (a) Total number of OTUs (in brackets) across all studied reactors found within each taxonomic order of the *Planctomycetia, Phycisphaerae*, and other minor classes. (b) Number of OTUs within each of the abundance categories: RT—rare taxa, CRT—conditionally rare taxa, and CRAT—conditionally rare and abundant taxa. (c) Fraction of OTUs within the set categories for each family level. (d) Total number of unique planctomycetotal OTUs detected across all samples, categorized by reactor type. (e) Number of OTUs in the individual sample per AD category. (f) Relative abundance of *Planctomycetota* (%) within the total bacterial community per reactor. The number of samples is indicated in brackets. (g) Visualization of PCoA based on the planctomycetotal OTUs Bray–Curtis distance matrix, coloured by the sampling location unit (‘U’).

Our earlier analysis of the anaerobic digester microbiota indicated that *Planctomycetota* comprise <1% of the community, categorizing them as part of the ‘rare microbiome’ (Calusinska et al. 2018). To gain in-depth perspective, we reanalysed the data with a specific focus on *Planctomycetota* OTUs (Supplementary File 1, Table S1). The ADs studied encompassed a wide range of operational conditions, processing various feedstocks as detailed in the ‘Methods’ section. In total, 376 OTUs were detected, primarily belonging to the *Planctomycetia* (*n* = 208) and *Phycisphaerae* (*n* = 122) classes (Fig. 1a). As only one *Ca*. Brocadiia OTU was assigned, we conducted an *in silico* PCR analysis to revise the specificity of the primer pair used (Supplementary File 1, Table S2). Our findings revealed that certain groups, including *Ca*. Brocadiia, were inadequately amplified, potentially leading to an underestimation of the true diversity of *Planctomycetota* in the AD reactors.

Since our dataset encompasses time series data, we could investigate the temporal dynamics of planctomycetotal OTU abundance within the AD microbial community. Therefore, we further split the planctomycetotal OTU dataset into three categories (Fig. 1b): rare taxa (RT; OTUs with a relative abundance below 0.01%), conditionally rare and abundant taxa (CRAT; OTUs between 0.01% and >1%), and conditionally rare (CRT; between 0% and 1%). As a result, 76.6% of the OTUs fell within the RT category (Fig. 1b), and primarily belonged to the *Planctomycetia* class (173 OTUs), as well as to other classes such as BD7-11, and vadinHA49 (Fig. 1c). In turn, *Phycisphaerae* OTUs assigned to the CCM11a, mle1-8, SG8-4, KCLunmb-38–53 and Pla1 families were classified as CRT. We detected only two OTUs that were placed within the CRAT category, comprising the Pla3 lineage and *Phycisphaerae* (KCLunmb-38–53) class.

Compared to the samples from OFMSW reactors, where the mean abundance (0.01% ± 0.02%) and richness (9 ± 3 OTUs) of *Planctomycetota* was minor, the highest diversity was observed in reactors treating sewage sludge and agricultural residues (Fig. 1d–f). On average, 52 ± 13 distinct OTUs were detected per sample in WWTP ADs, while 37 ± 20 were found in farm ADs (Fig. 1e). However, the highest number of unique *Planctomycetota* OTUs was found in farm reactors (Fig. 1d; 247 OTUs), possibly attributed to the broader range of feedstocks. The introduction of *Planctomycetota* from the gut environment, through manure used in farm reactors and human excreta processed in WWTP ADs, likely contributes to the high diversity of these bacteria in these types of AD systems. Additionally, the lower diversity in the OFMSW reactor could result from this reactor being represented by only one sampling location, while farm and WWTP samples were taken from three locations. The principal coordinate analysis (PCoA) clearly separated the planctomycetotal communities from farm and WWTP ADs (Fig. 1g), although farm ADs were much more dispersed and clustered according to the sampling location (ANOSIM test; *R* = 0.93, *P* < .01).

### Comparison of OTU sequences with described type strains of *Planctomycetota*

**Figure 2.**
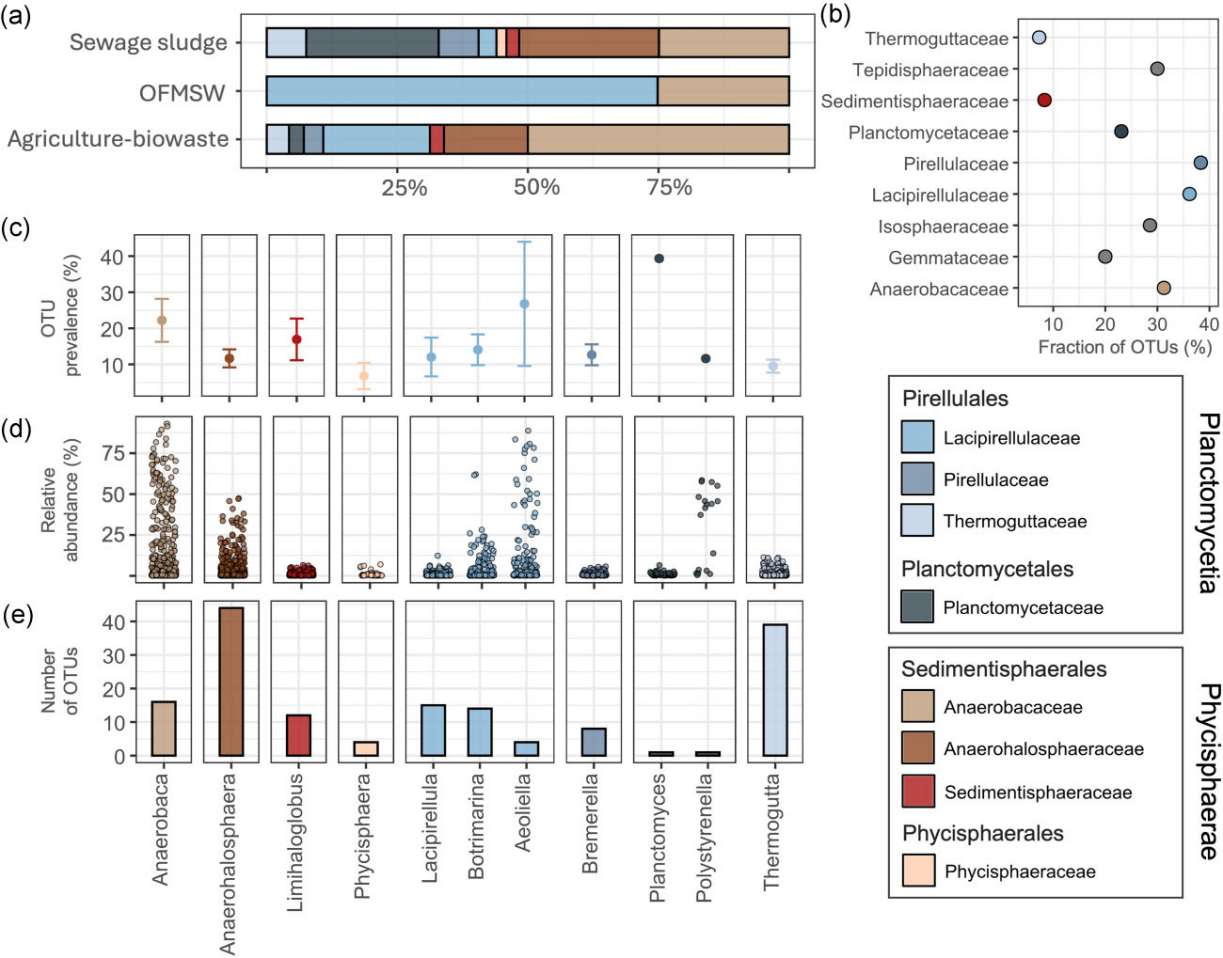
Phylogenetic relationship of AD *Planctomycetota* with previously isolated strains, coloured by family level as indicated in the legend. (a) Relative abundance (%) of the closest cultured relatives to the AD OTUs, grouped by family level and displayed for different reactor categories. (b) Fraction of OTUs likely falling within genera characterized by cultured representatives, shown for each family level. (c) OTU prevalence in AD reactors, e.g. fraction (%) of reactors containing assigned OTUs, displayed by genus level. (d) Relative abundance (%) of OTUs per sample within the planctomycetotal community. (e) Number of OTUs assigned to the listed genera across all the samples. In panels (b–d), only the most abundant genera are shown (>1% of planctomycetotal relative abundance). All records are detailed in Supplementary File 1.

To compare the phylogenetic relationship of AD *Planctomycetota* with previously isolated strains, we conducted a BLAST search against a database containing full-length 16S rRNA gene sequences from type strains of *Planctomycetota*. As a result, abundant AD OTUs were closely affiliated with three orders of *Planctomycetota: Sedimentisphaerales* from the *Phycisphaerae* class, and *Planctomycetales* and *Pirellulales*, both belonging to the *Planctomycetia* class (Fig. 2a). The closest relatives attributed to the *Pirellulales* were *Lacipirellulaceae, Pirellulaceae*, and *Thermoguttaceae* families, representing Pir4, p-1088-a5_gut_group, and ‘uncultured’ lineages in the SILVA database. Among OTUs with the highest similarity to the *Sedimentisphaerales* strains (MSBL9 in SILVA database), all the known families were covered, including *Anaerobacaceae* (previously SG8-4), *Anaerohalosphaeraceae*, and *Sedimentisphaeraceae* (Fig. 2a).

We further assessed the potential for isolating new *Planctomycetota* from ADs by applying the taxonomic thresholds defined by Yarza et al. (2014), which are based on 16S rRNA gene sequence identities (Yarza et al. 2014). Thresholds of 86.5%, 94.5%, and 98.7% sequence similarity were assigned at the family, genus and species levels, respectively. Importantly, although 16S rRNA gene sequences may be highly similar or nearly identical, they can still represent different species, indicating that the applied approach may lack the sensitivity to detect these differences. In the AD systems studied, the highest potential for new genera discovery was evidenced for the *Sedimentisphaeraceae* (8.3%) and *Thermoguttaceae* (7.3%) families (Fig. 2b). The threshold for the species level was passed by only 11 OTUs, mostly belonging to the transient community, and occurred in only a few samples of diverse reactors over the year (Supplementary File 1, Table S1). However, one OTU (Otu3705) assigned to *Planctomyces bekefii* with 99% identity was found in nearly 40% of all the samples. This OTU was regularly present throughout the year in one farm and all WWTP reactors. Since the sequencing used only allowed for the recovery of partial 16S rRNA gene sequences, further validation with full-length sequences is recommended to ensure more accurate taxonomic resolution.

In farm ADs (agriculture-biowaste), OTUs with the highest relative abundance were closely related to the *Anaerobaceae* family (Fig. 2a and c–e). Conversely, dominant OTUs in WWTP ADs were closely associated with *Planctomycetaceae*. At the genus level, the largest number of OTUs was closely related to the *Anaerohalosphaera* (44 OTUs) and *Thermogutta* (39 OTUs) isolates (Fig. 2e). However, *Thermogutta* appeared neither prevalent (Fig. 2c) nor abundant (Fig. 2d). Members of *Planctomyces* and *Polystyrenella* (both *Planctomycetaceae*) were each assigned to a single OTU (Fig. 2e). Interestingly, a *Polystyrenella*-assigned OTU (Otu616, Pla3 lineage according to the SILVA) was notably abundant in WWTP AD, constituting ~50% of the planctomycetotal relative abundance (previously identified as CRAT).

### The overall metabolic repertoire of *Planctomycetota* in comparison to other AD bacteria

**Figure 3.**
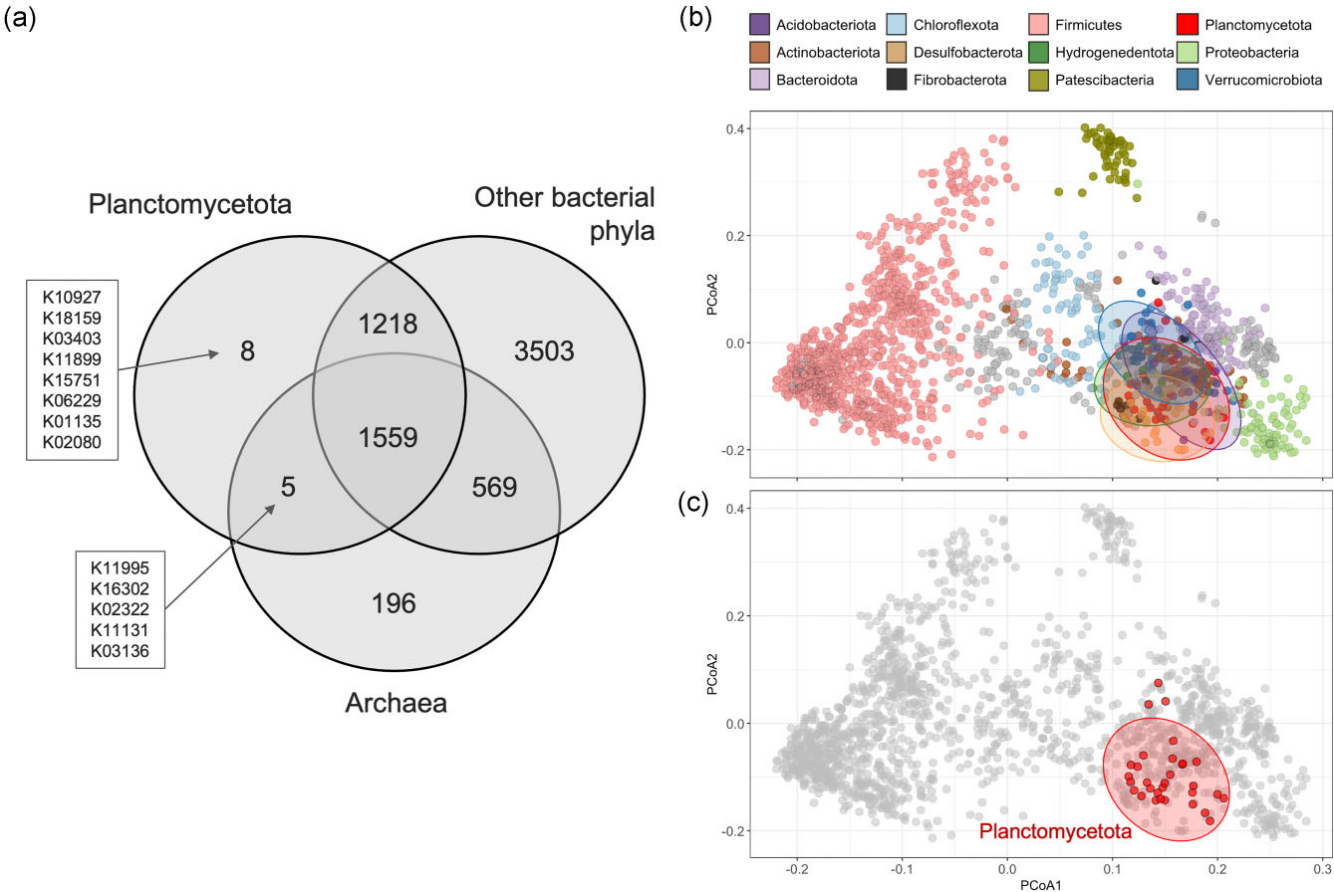
General encoded metabolic potential of *Planctomycetota* compared to other bacteria and archaea. (a) Venn diagram showing the number of shared and unique KOs. (b) PCoA plot comparing the presence–absence of KOs across the different bacterial genomes coloured by phylum taxonomic level. The plot includes ellipses representing 95% confidence around the group centroids and only the phyla with >90% ellipse intersections with *Planctomycetota* are coloured: *Acidobacterota, Hydrogenedentota, Desulfobacterota*, and *Verrucomicrobiota*. (c) The same PCoA plot highlighting the *Planctomycetota* MAGs only.

Our first step towards cultivating *Planctomycetota* was to highlight their unique metabolic functions utilizing all the MAGs from the study by Campanaro et al. (2020). Based on KEGG annotation, the *Planctomycetota* genomes share more genes common to both archaea and bacteria (1559) than those exclusively shared with bacteria (1218; Fig. 3a). KOs common for *Planctomycetota* and archaea encode proteins related to cell structure, RNA modifications, and transcriptional regulation (Supplementary File 2, Table S4). Unsurprisingly, *Planctomycetota* largely lack gene-encoding cell division proteins like *ftsZ* and *ftsE*, as well as several genes associated with cell growth (*recF, pbpA, udk*, and *rnhB*). Analysing the presence–absence gene profiles, the metabolic repertoire encoded by *Planctomycetota* shows significant overlap with *Acidobacterota, Hydrogenedentota, Desulfobacterota*, and *Verrucomicrobiota*, distinguishing them clearly from representatives of the *Bacillota* (formerly *Firmicutes*) and *Ca*. Patescibacteria genomes (Fig. 3b and c).

**Figure 4.**
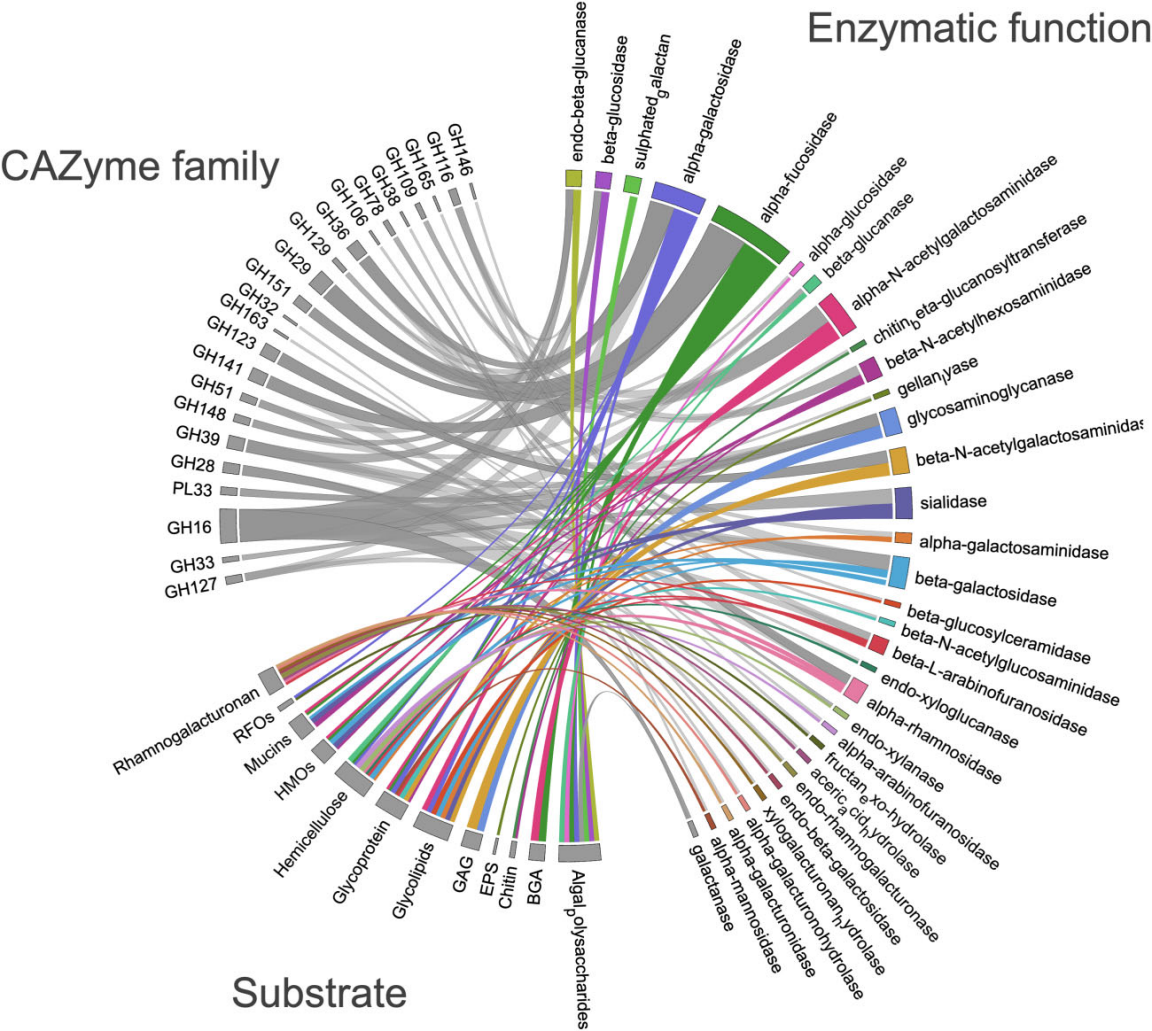
Putative substrates specific for *Planctomycetota* based on the genomic capacity for targeting glycosidic linkages. Lysosomal and other minor activities including oligosaccharidases are omitted. RFO—raffinose family oligosaccharides, HMO—human milk oligosaccharides, GAG—glycosaminoglycans, EPS—extracellular polymeric substances, and BGA—blood group antigen.

The ability to utilize diverse, and sometimes complex carbon sources, is advantageous in biomass-rich environments, such as AD. Therefore, we tried to predict the potential of *Planctomycetota* to efficiently acquire and metabolize the carbohydrates not commonly targeted by the other AD microbes. We also considered that such an analysis would help us select potential carbon substrates for the further isolation of *Planctomycetota*. We revealed that most of the unique (i.e. differentially encoded) CAZyme families in the genomes of *Planctomycetota*, encompass enzymes that act primarily on the terminal ends of polysaccharides and oligosaccharides (exo-acting, exoglycosidases). As such, *Planctomycetota* encode putative sialidases (GH33 and GH156), α-l-fucosidases, α-galactosaminades, or α-l-rhamnosidases (GH29, GH95, GH109, and GH151) differentially, which equips them with plausible enzymatic activities relating to the terminal residues of sialic acid, fucose, rhamnose, galactose, and *N*-acetyl-galactosamine/glucosamine (Supplementary File 2, Table S4). These sugar residues are commonly found in a range of diverse substrates including glycoconjugates (animal and human glycoproteins, glycolipids), glycosaminoglycans (GAGs), certain rhamnogalacturonans, and hemicelluloses or algae-derived polysaccharides (Fig. 4). Regardless of this, most *Planctomycetota* genomes also encodes for the CAZymes targeting lignocellulose such as GH5 or GH10. The ability to source carbon and energy from these distinct substrates indicates a specific niche for *Planctomycetota* in ADs, provided these substrates are present in the reactor.

### Validation of the preference of *Planctomycetota* for algal biomass

*Planctomycetota* is one of the phyla with the highest number of distinct algae-targeting CAZymes, which places it as bacteria with a conceivable capacity to utilize algal biomass (Supplementary File 4, Fig. 1). Moreover, certain *Planctomycetota* (*Pirellulales*) encode a number of genes for S1 family sulphatases (Supplementary File 2, Table S3) and sulphate uptake, which could help them compete with sulphate-reducing bacteria that will feed on the released sulphates from algal biomass. Therefore, to validate the preference of *Planctomycetota* for algal biomass experimentally, we attempted to enrich their community directly during the AD process. We performed BMP tests with cyanobacterial, macroalgal, and plant lignocellulosic biomasses using the WWTP AD sludge inoculum. Despite the overall well-encoded metabolic capacities of *Planctomycetota* to utilize different fractions of brown algal biomass, they did not survive the testing conditions in our BMP tests. Instead, they showed an increased abundance only in bottles supplemented with lignocellulosic biomass (Supplementary File 4, Fig. 2), suggesting their preference for cellulose and hemicellulose fractions, rather than algae-derived glycans. However, in this preliminary test, we only evaluated the brown algal biomass, and further experiments utilizing red or green algae should be undertaken to uncover the plausible algalytic potential of AD *Planctomycetota*. The bacteria that became dominant in algal biomass supplemented BMPs were predominantly *Bacillota, Bacteroidota*, and *Pseudomonadota* (previously *Proteobacteria*), which were likely engaged in the degradation of both cyanobacterial and macroalgal biomasses.

### Detailed analysis of the metabolic characteristics of *Planctomycetota* in anaerobic digesters

To further characterize *Planctomycetota* in AD reactors, we created own, more comprehensive database of *Planctomycetota* genomes, comprising MAGs used in our previous study (Klimek et al. 2024), supplemented with the newly reconstructed genomes from this study (Supplementary File 2, Table S3). The final database contained 39 nonredundant high-quality AD *Planctomycetota* genomes. It included 14 genomes from the *Pirellulales* order within the *Planctomycetia* class, 21 genomes assigned to the *Phycisphaerae* class, comprising 11 from the *Sedimentisphaerales* order and six from the UBA1845 order, along with three genomes from other classes (*Ca*. Brocadiia, putative UBA8108, and PUPC01 groups). Despite the abundance of *Planctomycetales* in the WWTP ADs, no MAGs were reconstructed and therefore we were unable to include them in the functional analysis. Importantly, on average, only about 37% ± 6 of the predicted genes are functionally annotated in the retrieved planctomycetotal genomes.

**Figure 5.**
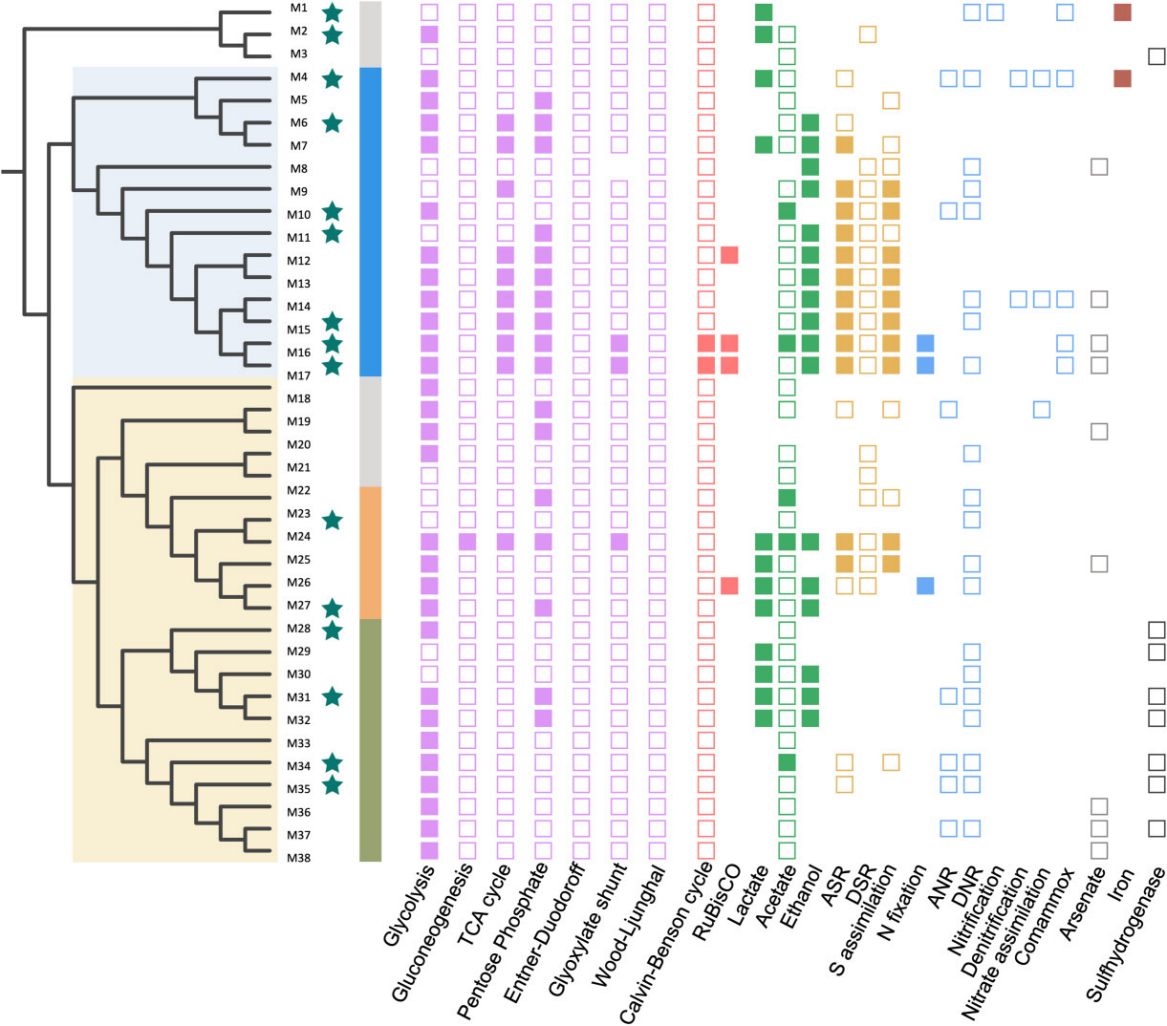
Metabolic reconstruction of AD *Planctomycetota* MAGs assembled in this study, including isolated/enriched planctomycetotal strains (M10, M16, and M17). The clade colour on the tree denotes taxonomic affiliation (*Planctomycetia* in blue, *Phycisphaerae* in yellow), with stars indicating MAGs reconstructed from wastewater ADs. The following colour strip designates MAGs taxonomy and the planctomycetotal orders: *Pirellulales* in blue, UBA1845 in orange, *Sedimentisphaerales* in green, and the other minor orders in grey. Square symbols represent metabolic traits based on the genomic annotation: fully coloured symbols indicate complete pathways (all required genes present, or only one is missing), empty symbols denote the partially encoded pathways (some required genes missing), and missing symbols indicate that no genes related to the pathway were found. Abbreviations: ASR—assimilatory sulphate reduction, DSR—dissimilatory sulphate reduction, ANR—assimilatory nitrogen reduction, and DNR—dissimilatory nitrogen reaction. Further details on the prediction of functional traits can be found in Supplementary File 2, Table S3.

To uncover the specific metabolic capabilities of the AD *Planctomycetota*, our analysis focused on genes involved in the carbon (C), nitrogen (N), and sulphur (S) metabolism, as well as in hydrogen production and utilization (Fig. 5). Analysis of the central carbon metabolism in the retrieved genomes suggests that most *Planctomycetota* likely generate energy through glycolysis and are capable of fermenting carbohydrates (Fig. 5). The majority of the *Planctomycetota* genomes retrieved (70%) encode for core set of flagella proteins, suggesting that they can be motile (Supplementary File 2, Table S3). Most *Pirellulales* seem to be capable of sulphate assimilation via the assimilatory sulphur reduction pathway (ASR), and this is frequently coupled with their ability to acquire elemental sulphur (Fig. 5). Most of the planctomycetotal genomes encode an almost-complete pathway for dissimilatory nitrate reduction to ammonium and a few representatives encode a complete nitrogenase complex allowing them to assimilate molecular nitrogen (M16, M17, and M26). In addition to the common bacterial [NiFe] and [FeFe] hydrogenases, [NiFeSe] hydrogenases are also encoded by 30% of planctomycetotal genomes (Supplementary File 2, Table S3). Nine planctomycetotal genomes encode *arsB* and *arsR* genes for arsenate reduction, but lack *arsC*, suggesting possible resistance to arsenate with a compromised reduction capacity. Iron oxidation and reduction operons are fully encoded by only two *Planctomycetota* from *Pirellulaceae* (M1) and UBA8108 (M4), respectively, while genes related to iron transport and acquisition are encoded by virtually all the genomes (Supplementary File 2, Table S3). Interestingly, we also noticed that some *Planctomycetota* genomes (M12, M16, M17, and M26) encode ribulose-1,5-bisphosphate carboxylase/oxygenase (RuBisCO) suggesting their capacity for autotrophic carbon fixation (discussed below).

### Genome-guided enrichment and isolation of *Planctomycetota*

To isolate *Planctomycetota* strains from the AD microbial community, we developed a list of media tailored to the encoded metabolic capacities and nutritional requirements of AD *Planctomycetota* MAGs. We set up 190 different enrichment conditions with various carbon, nitrogen and sulphur sources (Supplementary File 3, Table S7). We expected the success of the enrichments with added FtsZ stabilizers, given that *Planctomycetota* do not produce the FtsZ protein and in addition, we anticipated positive outcomes from the enrichments with specific carbohydrates. However, it is important to note that only one enrichment containing FtsZ stabilizers significantly enhanced *Planctomycetota* abundance (see below), while all other cultures treated with stabilizers did not promote similar growth suggesting either stochastic effects or other unresolved factors.

**Figure 6.**
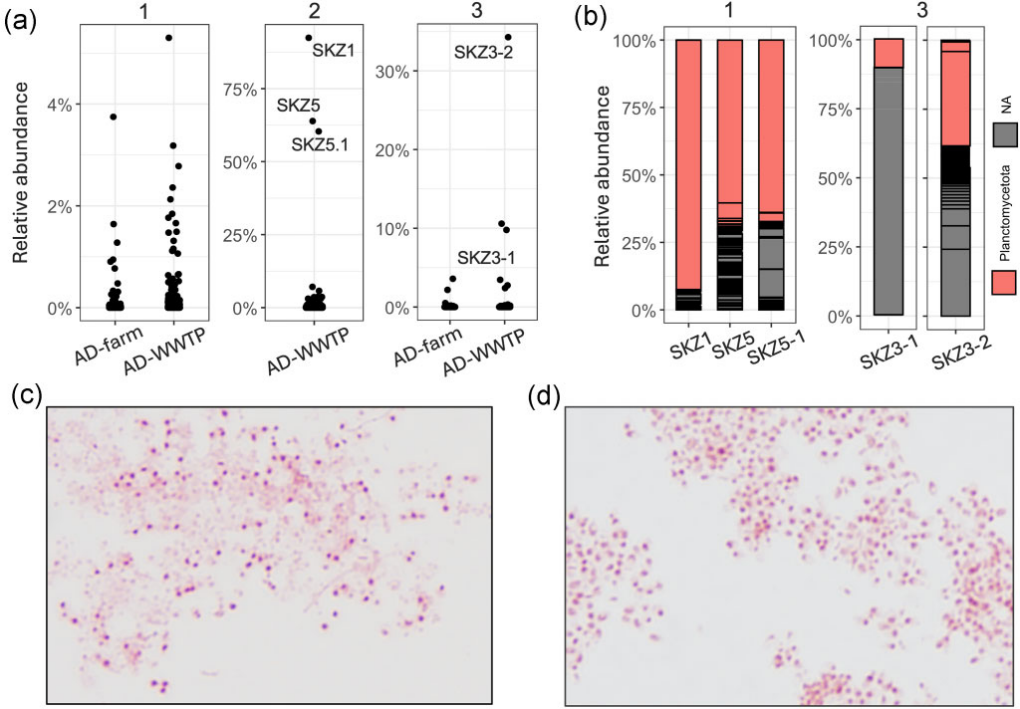
Enrichment and isolation of *Planctomycetota*; 1, 2, and 3 designate different inocula used. (a) Relative abundance of *Planctomycetota* in all the enrichments in our study. (b) Community analysis at the end of the incubation period, with each bar representing a single OTU; only the significantly enriched samples on mucin, fucoidan, and NAG substrates are shown. (c) Gram-stained microscopy image of the purified SKZ1R strain isolated from the SKZ1R enrichment. (d) Gram-stained microscopy image of the SKZ5-1 enrichment, *Pirellula*-like shape cells are captured.

At the end of the incubation period, we analysed the selected enrichments using 16S rRNA gene sequencing and excluded those where *Planctomycetota* were not present (Supplementary File 3). Although most of the WWTP sludge inoculated enrichments did not show a significant growth of *Planctomycetota* (Fig. 6a), we successfully enriched a few *Planctomycetota* cultures and succeeded in isolating one strain from the enrichment SKZ1R axenically by the end of the experiment (Fig. 6b). Initially, all enriched/isolated strains constituted <1% in the microbial community abundance in the inoculum sludge. The SKZ1R enrichment was first treated with antibiotics (ampicillin, vancomycin, and streptomycin) and FtsZ stabilizers (2,6-difluorobenzamide and PC190723) for 2 weeks, and then recultured on a medium containing *N*-acetyl-glucosamine as the sole carbon and nitrogen source. After 2 weeks of incubation, 16S rRNA gene analysis revealed that a single planctomycetotal OTU dominated, accounting for 94% of the relative abundance (Fig. 6b). We then purified the strain over two continuous passages. Microscopic inspection revealed the Gram-negative ‘*Pirellula*-like’ shape of the SKZ1R culture, which aggregated in the medium as faintly grey flocs (Fig. 6c). Unfortunately, subsequent passages became contaminated by spore-forming bacteria, necessitating further isolation trials to purify the strain. Despite this, we managed to reconstruct its genome and gained insights into the functional potential of this new bacterium.

Enrichments containing porcine-extracted mucin as the sole carbon source were also very effective for enriching *Planctomycetota*. Three cultures SKZ5, SKZ5R, and RCS-4 (Fig. 6a; Supplementary File 3, Table S9) showed significant growth of four *Pirellulaceae* species with a reduced diversity of other bacteria such as *Bacteroidota* (Supplementary File 3, Table S9). Microscopic inspection also revealed a *Pirellula*-like shape for bacteria in the SKZ5R culture (Fig. 6d). Despite the high abundance of *Pirellulales* representatives in these enrichments (35%, 60%, and 63% of relative abundance), we were unable to isolate these bacteria axenically using various cultivation methods. In turn, the SKZ3 enrichment was performed using commercially available fucoidan from *Undaria pinnatifida*, as the primary carbon source. During the initial enrichment phase, e.g. after 1 month, a single OTU assigned to *Lacipirellulaceae* constituted ~5% of the relative abundance. However, this abundance increased significantly to nearly 40% in the subsequent 2-year period and was maintained with regular feeding and only two culture passages (Fig. 6b).

### Proposition of ‘*Ca*. Luxemburgiella decessa’

**Figure 7.**
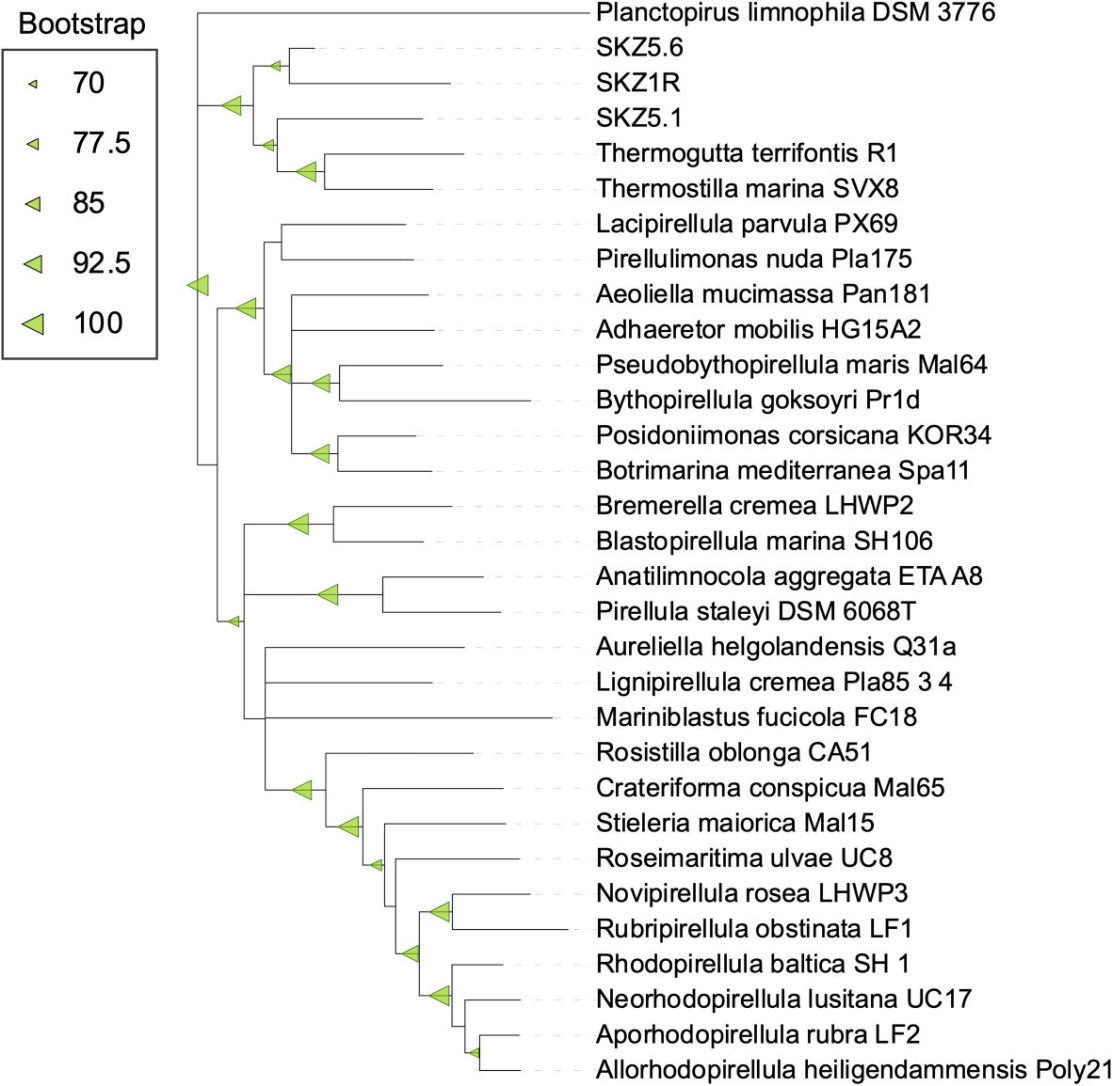
16S rRNA phylogeny of SKZ strains and type strains of *Pirellulales*.

Isolated and enriched strains from the SKZ1R and SKZ5 cultures (further called ‘SKZ strains’) were sequenced and their genomes were reconstructed. This resulted in the genome of the isolated SKZ1R strain being obtained, as well as two genomes from the SKZ5 enrichment, designated as SKZ5.1 and SKZ5.6. Phylogenetic analysis based on the 16S rRNA tree, including type species of the *Pirellulales* order, indicated that all enriched strains clustered within the *Thermoguttaceae* family with strong bootstrap support (Fig. 7). Subsequently, we performed a BLAST search using the RNA gene sequences of all isolated/enriched OTUs against the SILVA v138 database, considering hits with >98.7% similarity (Supplementary File 2, Table S5). We did not find any sequence matches for the SKZ1R and SKZ5.1 strains, whereas for the partial 16S rRNA sequence of SKZ5.6, we identified two hits with 100% identity to bacteria found in subsurface aquifer sediment and rice paddy soil.

We calculated the ANI and AAI of these SKZ strains in relation to the closest type species, *T. terrifontis*, and constructed a phylogeny with all MAGs assigned to the *Thermoguttaceae* family retrieved from the GTDB database (Supplementary File 4, Fig. S3). Based on the 16S rRNA phylogeny, rpoB gene as well as ANI, and AAI thresholds (Table 1), we propose that SKZ1R and SKZ5.6 represent two species within the same genus, while SKZ5.1 represents another genus within the *Thermoguttaceae* family. Due to the loss of viability of the SKZ1R strain and lack of purified cultures for the SKZ5 strains, we propose describing only the SKZ1R strain as a *Candidatus* status, as it meets the criteria suggested for describing novel taxa of uncultivated prokaryotes (Konstantinidis et al. 2017). Therefore, we propose a novel genus*, Ca*. Luxemburgiella gen. nov., within the *Thermoguttaceae* family, *Pirellulales* order, and *Planctomycetia* class of the *Planctomycetota* phylum.

**Table 1.** Comparison of 16S rRNA, rpoB, ANI, and AAI similarities between SKZ strains and type species and closest relatives of the *Thermoguttaceae* family: *Thermogutta terrifontis* R1 (T), *Thermogutta hypogea* SPB2, and *Thermostilla marina* SVX8 (T). Below the related genomic information of isolated/enriched SKZ strains and type species, *T. terrifontis* R1.

		R1 (T)	SBP2	SVX8 (T)	SKZ1R	SKZ5.6	SKZ5.1
16S RNA	SKZ1R	88.0	88.4	88.8		94.3	87.4
	SKZ5.6	93.9	94.3	92.6	94.3		88.0
	SKZ5.1	88.1	90.6	89.0	87.4	88.0	
ANI	SKZ1R	54.3				77.4	55.9
	SKZ5.6	54.6			77.4		
	SKZ5.1	52.9			55.9		
AAI	SKZ1R	67.9				79.4	70.1
	SKZ5.6	67.6			79.4		
	SKZ5.1	67.5			70.1		
rpoB	SKZ1R	76.2				94.3	80.4
	SKZ5.6	76.8			94.3		
	SKZ5.1	76.8			80.4		
Genomic metadata	Genome size (Mb)	4.8			10.2	8.5	5
	Number of genes	4559			7708	6644	3543
	GC%	57.5			63.1	62	63.7
	Completeness (%)	96.9			98.9	100	96.6
	Contamination (%)	2.2			2.3	0	0

*Candidatus* Luxemburgiella gen. nov. (Lu.xem.bur.gi.el'la. N.L. fem. n. Luxemburgiella, named after Luxemburg, the location of the anaerobic digester from which the type species was isolated).

At present, *Ca*. Luxemburgiella encompasses one species:

*Candidatus* Luxemburgiella decessa SKZ1R(T) sp. nov. (de.ces'sa. L. fem. part. adj. decessa, withdrawn, pertaining to lost purified culture).

Strain SKZ5.6 represents another species within the *Ca*. Luxemburgiella genus, but due to the lack of purified culture, we refrain from naming the bacterium at this time.

### Insights into the metabolic potential of isolated *Thermoguttaceae* strains

Based on the genome analysis, strains SKZ1R and SKZ5.6 exhibit remarkable versatility in their metabolic potential. These strains might generate energy putatively through various pathways, supporting robust biosynthesis and energy conservation mechanisms (Fig. 5; M16 and M17). Despite their low abundance, their ability to assimilate nitrogen and sulphate suggests they play a role in the N and S cycles within the AD environment. It is likely that both strains are able to utilize a broad range of complex carbohydrates (Supplementary File 2, Table S6), indicating their flexibility in carbon source utilization and involvement in organic matter degradation.

As the SKZ5.1 and SKZ5.6 strains grew in a mucin-based medium, we explored their genomes further for genes related to protein utilization and sialic acid metabolism. SKZ5.1 strain encodes over 80 copies of sialidase genes (GH33 and GH177), along with a sialic acid transporter (NanT) and *N*-acetylamannosamine kinase (NanK) but lacks the *N*-acetyleneuraminate lyase (NanA) needed for the critical step of converting *N*-acetylneuramine to pyruvate and *N*-acetylmannosamine. In turn, SKZ5.6 encodes a complete Nan cluster, including sialidases, sialic acid transporter (NanT), *N*-acetylneuraminate lyase (NanA), and *N*-acetylmannosamine kinase (NanK), indicating its capacity to utilize sialic acids. However, based on the gene search against the MEROPS database, neither strain encodes known metalloproteases specialized in cleaving the protein backbone of glycoproteins, including mucins.

Interestingly, SKZ1R and SKZ5.6 strains appear to fix carbon autotrophically, based on the presence of a nearly complete Calvin cycle, including the large subunit of RuBisCO (K01601). This, along with the glycolysis, tricarboxylic acid, and pentose phosphate pathways, allows for the simultaneous utilization of organic and inorganic carbon sources, making them mixotrophic. The two encoded bidirectional hydrogenase complexes suggest that CO_2_ fixation could be coupled with hydrogen oxidation. We also searched for the presence of RuBisCO in other AD MAGs and evidenced that the small (K01602) and large (K01601) subunits of RuBisCO were not very common. The K01601 subunit was mainly found in archaea (80%) and *Acidobacteria* (83%) genomes, while the K01602 subunit was encoded in only a few phyla, including six *Pseudomonadota* genomes (7%) that contain both K01601 and K01602 subunits. Based on the NCBI Conserved Domain Database sequence analysis, the planctomycetotal RuBisCO protein belongs to Form I, which is mainly detected in *Pseudomonadota* (Badger and Bek 2008).

## Discussion

### Most of the *Planctomycetota* OTUs represent rare taxa within the AD community

Our study reveals that the *Planctomycetota* in the AD microbial community are characterized by numerous rare OTUs (Fig. 1). The core community comprises only a few prevalent OTUs, primarily within three main orders: *Pirellulales, Planctomycetales*, and *Sedimentisphaerales*. In particular, the *Planctomycetales* order includes a high diversity of OTUs (Fig. 2). Despite their low individual abundances, collectively, these OTUs represent a large portion of *Planctomycetota* diversity in the WWTP ADs, suggesting a reservoir of genetic and functional potential. However, due to the lack of reconstructed genomes for *Planctomycetales*, we were unable to explore their putative functional capacities in AD further.

While little is known about the two groups assigned as CRAT taxa, Pla3 and KCLunmb-38–53 groups, the latter has been identified as hydrolytic and acidogenic bacteria that become more abundant in reactors treating food waste (Peng et al. 2023). Based on the SILVA database, there are 129 nonredundant 16S rRNA gene sequences belonging to the Pla3 lineage, retrieved from diverse environments including marine, freshwater, and anoxic habitats, indicating that these enigmatic bacteria is widespread. CRATs are known to fluctuate between rarity and abundance in response to environmental shifts, indicating their potential role in maintaining ecosystem stability and resilience (Shade et al. 2014). The limited number of CRATs observed in our study could be due to the sampling being undertaken in largely stable environments with few disturbances to trigger fluctuations or could reflect specific ecological niches where these taxa operate. Future metatranscriptomic approaches could provide deeper insights into the metabolic capabilities and activity patterns of these bacteria.

Microbial community studies have shown that the *Sedimentisphaerales* order (MSBL9) is found predominantly in sulphur or methane-rich anoxic marine sediments, hypersaline lakes, or deep sea habitats (Hamdan et al. 2018, Spring et al. 2018). However, the SILVA database reveals an immense diversity within MSBL9, and only certain groups affiliated with this order were detected in our study. Among *Pirellulales*, the *Pirellulaceae* family is distributed globally in marine environments, often strongly associated with different macroalgae (Lage and Bondoso 2011, Vitorino and Lage 2022). In contrast, the *Thermoguttaceae* family is typically found in low-oxygen habitats, including hydrothermal vents, deep sea sediment, and gut microbiome (Slobodkina et al. 2015), and while *Lacipirellulaceae* representatives are predominantly isolated from marine or brackish environments, certain lineages have also been found in low-oxygen aquatic environments, including ADs (Dedysh et al. 2020).

### AD-sourced *Planctomycetota*: a metabolically diverse group comparable to their closest characterized relatives from other environments

*Planctomycetota*, along with other low-abundant phyla such as *Armatimonadota* and *Hydrogenedentota* are attributed the highest carbohydrolytic potential in the AD environment (Campanaro et al. 2020, Vanwonterghem et al. 2016), and are considered important primary degraders in other environments (Ivanova et al. 2018, Wang et al. 2015). Members of the *Sedimentisphaerales* order have been identified as initial degraders of particulate algal organic matter in carbohydrate-rich sediments of the Baltic Sea (Suominen et al. 2021), and it is suggested that they are capable of utilizing complex carbohydrates in a coal bed methane environment (Robbins et al. 2016). In turn, marine *Planctomycetota*, especially *Pirellulales*, likely utilize polysaccharides produced by diverse algae (Faria et al. 2018, Lage and Bondoso 2014, Ma et al. 2022). Despite the extensive hydrolytic potential, *Planctomycetota* often exhibit low abundance in carbohydrate-rich environments, including ADs, and many cultured strains within this phylum are slow growers (Lage and Bondoso 2012). Although the proliferation rate of different *Planctomycetota* members in the AD environment has not yet been extensively studied, they have been observed to decrease in abundance with reduced retention times (i.e. time that the organic material remains in the reactor for the digestion) (Krakat et al. 2011). Interestingly, some microbial species that initially appear to be slow growers, and are outcompeted by rapidly growing bacteria, are in fact fast growers with a delayed initiation of division (Buerger et al. 2012). Therefore, other ecological or physiological factors may also influence the abundance of *Planctomycetota*, making this area worth exploring to fully understand their dynamics and activity in this environment.

Our analysis indicates that members of the most prevalent taxa, especially the diverse *Pirellulales*, may be involved in nutrient cycling during the AD process, as suggested by their encoded metabolic traits (Fig. 5). Previously, *Planctomycetota* were highlighted as accelerators of methane production (Mustapha et al. 2018) and their abundance was correlated with ammonium nitrogen concentrations, likely due to their involvement in ammonia removal (Bellucci et al. 2022, Li et al. 2015a). Metagenomic studies have shown that diazotrophic *Planctomycetota*, along with *Pseudomonadota*, significantly influence marine nitrogen bioavailability in the open ocean (Delmont et al. 2018). Additionally, deep sea anammox *Ca*. Brocadiia are also assumed to oxidize iron (Schauberger et al. 2024). A positive correlation between flavins and MSBL9 suggests that this group might be engaged in pollutant breakdown, or iron oxidization in contaminated areas and anoxic sediments (Monteverde et al. 2018).

While much research on the microbial metabolic potential has focused on the phylum level (Campanaro et al. 2020), our study reveals significant diversity in metabolic capacities among planctomycetotal members, as evidenced by the strains isolated in this study. *Ca*. Luxemburgiella strains exhibit potential metabolic plasticity, enabling them to adapt and survive in diverse conditions. During AD operations, microorganisms regularly encounter substrate limitations and drastic environmental changes (Khesali Aghtaei et al. 2022), prompting them to employ diverse strategies for survival. The ability to derive energy from both organic and inorganic sources clearly enhances their competitive advantage. Although nonanammox *Planctomycetota* have traditionally been considered chemoorganotrophic, MAGs assembled from seagrass bed sediments have been identified as carbon fixers via the Wood–Ljungdahl pathway (Chi et al. 2023), while those from cold seeps were shown to rather encode RuBisCO (Jiang et al. 2022), indicating a wider range of possible metabolic pathways. The SKZ strains, isolated in this study, represent species not yet detected in other environments, which suggest that they occupy specific niches within the AD system. While *Planctomycetota* are commonly detected, but still rare bacterial phylum in the AD microbiome, their presence may be attributed to their diverse metabolic capacities. Nevertheless, *in vitro* testing remains an important component in identifying these metabolic traits.

### Mucin-based cultivation approaches as a promising strategy for the enrichment of *Pirellulales* from WWTP ADs

The isolation process was highly challenging, and none of our enrichment attempts successfully promoted the growth of *Phycisphaerae*. Instead, our approaches were mostly biased towards isolating planctomycetial *Pirellulales*. This order has the highest number of axenically isolated species (Vitorino and Lage 2022), suggesting that they are more amenable to cultivation in the currently employed laboratory settings. Recently, a novel isolate of the SG8-4 group from *Sedimentisphaerales*, namely *Anaerobaca lacustris*, was isolated axenically (Khomyakova et al. 2024). We anticipate that this breakthrough, along with increased knowledge of this bacterium's phenotypic features, will enable us to isolate related strains from AD environments in the near future.

Despite extensive efforts and various approaches that were initially ineffective in cultivating *Planctomycetota*, we observed a promising trend to enrich *Pirellulales* in mucin-containing media (Fig. 6). This observation manifests the potential significance of these bacteria beyond the AD environment. Mucins are high molecular weight glycoproteins with distinct structures and functions, comprising a substantial fraction of the mucus layer in various regions of both human and animal bodies, such as the gastrointestinal tract, lungs, and oral cavity (McGuckin et al. 2015). Here, we used commercially available porcine-extracted mucin type III, which contains bound sialic acids at 0.5%–1.5% substitution. We hypothesize that, given the wide abundance of exoglycosidases targeting the sugar decorations present in mucins, such as sialic acids, the isolated SKZ strains could utilize this substrate as a carbon source. Sialic acid metabolism was once considered to be uniquely confined to the human pathogens and commensals, enabling them to exploit this abundant carbon source on mucus-rich surfaces, and is often associated with virulence (Almagro-Moreno and Boyd 2009). However, a recent analysis of microbial sialic acid catabolism has revealed its widespread occurrence in various nonhost associated environments, including engineered systems like ADs (Li et al. 2023). Moreover, metagenomic datasets have identified a wide diversity of GH156 sialidases in environmental *Planctomycetota*, although these enzymes likely target plant glycans (Mann et al. 2022).

While the ecological role of nonhost associated bacteria to encode for genes related to the sialic acid metabolism remains uncertain, it is reasonable to assume that the majority of WWTP AD microbiomes originate from the animal, mainly human gastrointestinal tract, which is the primary source of substances rich in sialic acids (Almagro-Moreno and Boyd 2009). Although the presence of *Planctomycetota* in the human gut is not well documented (Cayrou et al. 2013), various animal gut microbiomes have been shown to host members of this phylum, predominantly *Pirellulales* (Gallet et al. 2022, Gharechahi et al. 2022, Hu et al. 2024, Köhler et al. 2008). Despite increasing research into these bacteria, more comprehensive investigations are required to fully understand their potential for mucin utilization, especially concerning nutrient uptake systems. For instance, *Akkermansia muciniphila*, a hallmark member of the *Verrucomicrobiota* phylum, possesses a highly specialized metabolic system for exclusively utilizing mucin, which is not found elsewhere in the bacterial world yet (Ottman et al. 2017). This bacterium coordinates its transport system and enzymatic machinery to forage solely for glycoproteins from the mucous layer in the human gut (Davey et al. 2023). Consequently, it utilizes sialidases only as accessory enzymes for initial degradation of mucins, and shares this released monosaccharide with other sialic acids utilizing bacteria (Shuoker et al. 2023). It is important to note that all our mucin enrichments were cocultures, predominantly with *Bacteroidota*, which may introduce the possibility of cross-feeding among the enriched bacteria. Recently, a new marine *Planctomycetota, Rhodopirellula halodulae*, was described as capable of growing in a medium containing mucin as the sole carbon source (Pk et al. 2024). Although research into mucin use by the diverse members of *Pirellulales* is in its infancy, future endeavours using mucin-based cultivation approaches for isolation seem to be promising. We think that *Pirellulales* might represent a putatively important but currently overlooked group of the gut microbiome. As such, it might be beneficial to extend our focus from environmental to host-associated *Planctomycetota* lineages, as previously suggested by Kaboré et al. (2020).

### Key exoglycosidases for polysaccharides utilization as potential functional adaptability of *Planctomycetota*

The clear disappearance of *Planctomycetota* during the AD of both cyanobacterial and macroalgal biomass is intriguing. Despite *Planctomycetota* having a vibrant genetic capacity to encode for enzymes purely targeting the main polysaccharides of brown algal polysaccharides such as laminarins, fucoidans, or alginates, they were outcompeted by other bacteria in the BMP tests, despite their competitors being less versatile in their algalytic potential. Regardless, the *Planctomycetota* SKZ3 strain appeared to be highly enriched in the medium containing purified fucoidan, highlighting its niche specialization. As a phylum, *Planctomycetota* encompasses a wide encoded potential for fucoidanases and different strains have also been observed to utilize fucoidans (Gonzalez et al. 2024, Klimek et al. 2024). In a complex biomass, fucoidans may not be easily accessible, making it challenging for the well-equipped *Planctomycetota* to outcompete faster-growing and generalist bacteria that can utilize other fractions of the biomass. Previously, *Planctomycetota* were observed as one of the bacteria involved in protein hydrolysis and the fermentation of raw *Scenedesmus* sp. microalgae (Zamorano-López et al. 2019). However, it has also been suggested that the presence of *Planctomycetota* in ADs treating microalgal biomass was due to the carryover of satellite marine *Rhodopirellula* species that regularly coexist with microalgae (Li et al. 2015b). The AD of algae is a growing area of interest globally, although it has not yet been widely applied on the commercial scale (Thakur et al. 2022). Therefore, more research is needed to fully understand the processes involved, including a deeper investigation into the putative role and dynamics of *Planctomycetota*.

Interestingly, aside from the apparent CAZyme families, for which the enzymatic activities described indicate only the specificity for algae-derived carbohydrates, some might also be specific for other glycans and *vice versa*. For instance, the activity of enzymes belonging to the GH29 family relies on cleaving the terminal sugars of fucoses, which can be specific to both sulphated fucoidans and also mucins. Overall, the exoglycosidases that cleave the monosaccharides from their nonreducing ends can act on the diverse spectrum of substrates, including fucosylated, rhamnosylated and galactosylated glycans. These enzymes might exhibit broad specificity, making them useful for targeting a variety of polysaccharides and glycoconjugates, including various mucins, human milk oligosaccharides, glycosaminoglycans, hemicelluloses, pectins and finally, algal polysaccharides (Fig. 4). Indeed, previous studies have shown that diverse *Planctomycetota* utilized the GAGs such as chondroitin sulphates (Elshahed et al. 2007, Jeske et al. 2013, Reintjes et al. 2017), and the soil matrix of extracellular polymeric substances that resemble these substrates structurally (Wang et al. 2015). Chitin, a long-chain polymer of NAG and a primary component of the fungal cell walls and invertebrate exoskeletons, was also evidenced to be utilized by certain strains of *Planctomycetota* (Ravin et al. 2018, Wieczorek et al. 2019). This suggest that the ability to target diverse but structurally similar carbohydrates is a common trait of *Planctomycetota*, with specific abilities likely evolving in response to the particular niche they inhabit.

## Conclusions

Our study reveals that *Planctomycetota* in the AD microbial community are characterized by a high number of rare OTUs and only a few prevalent and abundant taxa. The core planctomycetotal community is restricted to three main groups: the *Pirellulales, Planctomycetales*, and *Sedimentisphaerales* orders. The origin of feedstocks and their chemical composition play a crucial role in shaping the structure of the planctomycetotal community. The highest diversity in the individual reactors is observed in ADs treating sewage sludge, while the highest number of unique OTUs was detected in farm reactors. Isolated strains from our study represent rarely occurring bacteria, belong to the *Thermoguttaceae* family, yet characterized by only three cultured species from other environments. The novel *Ca*. Luxemburgiella decessa encodes the metabolic traits for a mixotrophic lifestyle in its genome, highlighting the potential for discovering novel microbial species with unique metabolic features within this group. Although *Planctomycetota* is a low abundant group of bacteria in the AD environment, they might nevertheless be involved in the removal of sulphur and nitrogen species and degradation of various fractions of organic matter. The genomes of *Planctomycetota*, along with *Armatimonadota* and *Hydrogenedentota*, offer the widest CAZyme family repertoire, but also encode the most unique catalytic properties and substrate specificities among all the AD bacteria. Their exo-acting enzymes targeting fucose-, galactose-, and *N*-acetylglucosamine/galactosamine residues can be utilized for the remodelling of glycoproteins, including the mucus layer, or the digestion of certain algal polysaccharide fractions. Despite the encoded potential of *Planctomycetota* for macroalgal biomass degradation, they did not utilize it under the tested conditions. This demonstrates that *in vitro* testing remains an important component identifying metabolic traits. In this work, we outlined how genomic comparison can lead to informed testing of cultivation, highlighted by our successful enrichment of new species based on their nutritional requirements. However, further research is needed to elucidate the actual planctomycetotal activity and dynamics during the AD process. Overall, our findings underscore the significant presence and metabolic potential of *Planctomycetota* in methanogenic reactors, warranting further exploration and characterization of this enigmatic phylum.

## Supplementary Material

fiaf025_Supplemental_Files

## Data Availability

The raw sequencing data, reconstructed MAGs from metagenomic samples and genome sequencing data for the isolated strains have been deposited in GenBank under the accession number PRJEB79855. The 16S rRNA sequence of the proposed *Ca*. Luxemburgiella decessa SKZ1R is deposited under the accession number PV008844.
