# Supplementary material for: Insights into the phylogenetic and metabolic diversity of Planctomycetota in anaerobic digesters and the isolation of novel Thermoguttaceae species
Source: FEMS Microbiol Ecol. 2025 Mar 17;101(4):fiaf025. doi: 10.1093/femsec/fiaf025 (PMC11929135; doi:10.1093/femsec/fiaf025)
Supplement: fiaf025_Supplemental_Files [file fiaf025_supplemental_files.zip › Supplementary File 4.pdf]

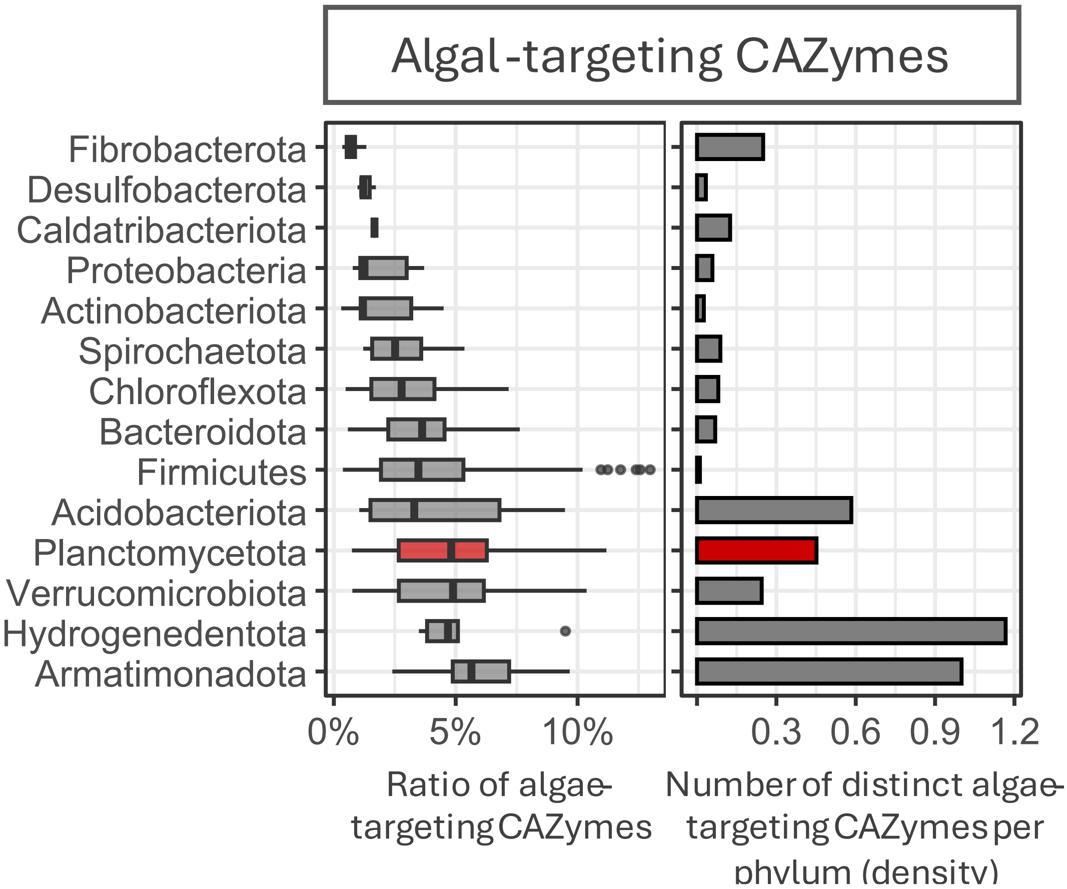

Fig. S1. Fraction of CAZymes targeting backbone of algal polysaccharides compared to all the detected CAZymes encoded in individual genomes gathered at the phylum level. Right panel: The CAZyme diversity factor e.g., number of distinct algae-targeting CAZymes detected in each phylum accounted for a different number of genomes.

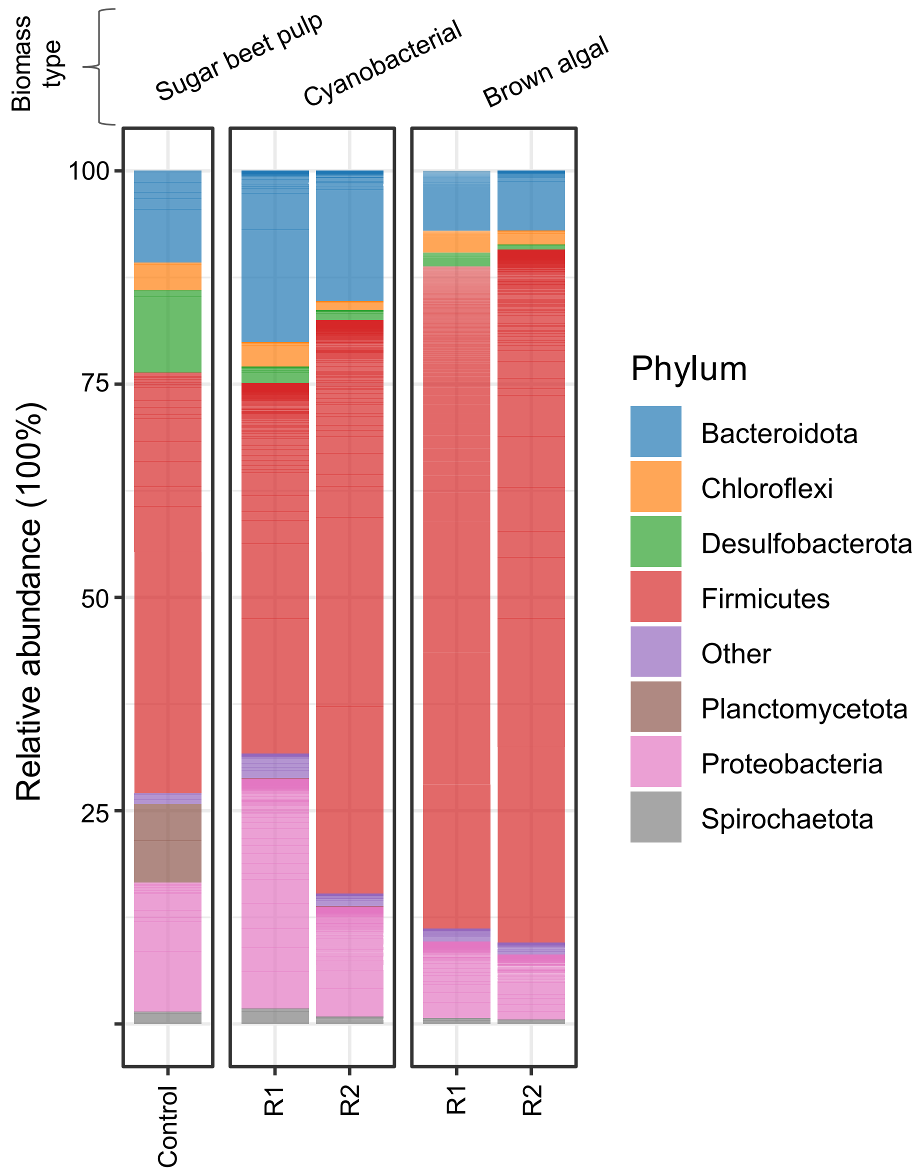

Fig. S2. The 16S rRNA gene sequencing results from the BMP test. R1 and R2 – replicate samples. In brown colour the *Planctomycetota* counts are highlighted.

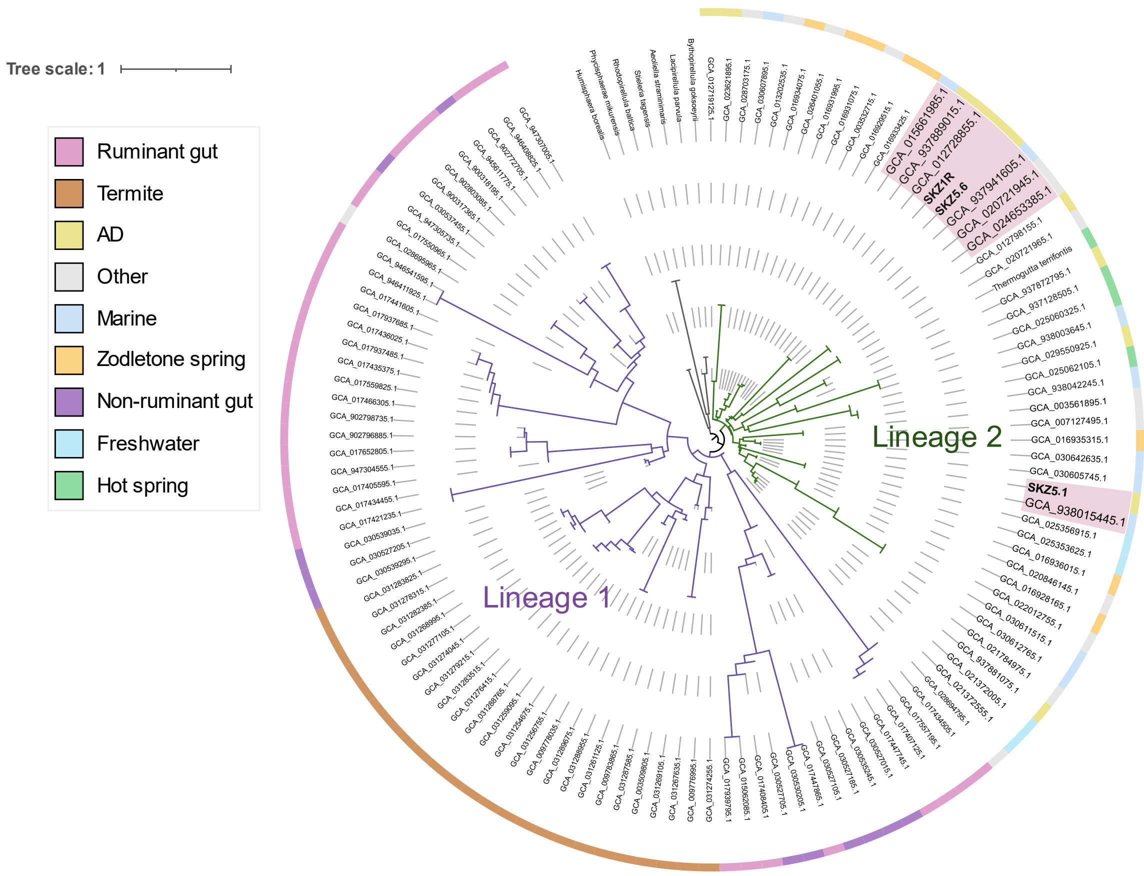

Fig. S3. *Thermoguttaceae* tree ecology context, performed for all the non-redundant *Thermoguttaceae* genomes in the GTDB database. The novel genera from the study and their closest metagenomic relatives branched are coloured in light pink. The outgroup for the tree construction included other species representing diverse *Planctomycetota*: *H. borealis, P. mikurensis, R. baltica, S. tagensis, A. straminimaris, L. parvula, B. goksoeyrii.* The majority of *Thermoguttaceae* MAGs were reconstructed from the animal gastrointestinal tract metagenomes and form a separate lineage ("Lineage 1") within this family. While a clear termite-derived cluster can be observed, ruminant and non-ruminant *Thermoguttaceae* seem to be more dispersed within the lineage. The SKZ strains are phylogenetically closely related to the sister lineage ("Lineage 2") that comprises genomes retrieved from various environments including marine, AD, and hot springs habitats.
